# MVT-Net: A novel cervical tumour segmentation using multi-view feature transfer learning

**DOI:** 10.1371/journal.pone.0325424

**Published:** 2025-06-24

**Authors:** Yao Yao, Yunzhi Chen, An Yang, Ye Ye, Lichun Wei, Shuiping Gou, Hua Yang

**Affiliations:** 1 School of Information Engineering, Hangzhou Vocational and Technical College, Hangzhou, Zhejiang, China; 2 Department of Radiation Oncology, the First Affiliated Hospital of Air Force Medical University, Xi’an, Shaanxi, China; 3 School of Artificial Intelligence, Xidian University, Xi’an, Shaanxi, China; Chungbuk National University, KOREA, REPUBLIC OF.

## Abstract

Cervical cancer is one of the most aggressive malignant tumours of the reproductive system, posing a significant global threat to women’s health. Accurately segmenting cervical tumours in MR images remains a challenging task due to the complex characteristics of tumours and the limitations of traditional methods. To address these challenges, this study proposes a novel cervical tumour segmentation model based on multi-view feature transfer learning, named MVT-Net. The model integrates a 2D global axial plane encoder-decoder network and a 3D multi-scale segmentation network as source and target domains, respectively. A transfer learning strategy is employed to extract diverse tumour-related information from multiple perspectives. In addition, a multi-scale residual blocks and a multi-scale residual attention blocks are embedded in the 3D network to effectively capture feature correlations across channels and spatial positions. Experiments on a cervical MR dataset of 160 images show that our proposed MVT-Net outperforms state-of-the-art methods, achieving a DICE score of 75.9±7.43%, an ASD of 2.69±0.58 mm and superior performance in tumour localisation, shape delineation and edge segmentation. Ablation studies further validate the effectiveness of the proposed multi-view feature transfer strategy. These results demonstrate that our proposed MVT-Net represents a significant advance in cervical tumour segmentation, offering improved accuracy and reliability in clinical applications.

## 1 Introduction

Cervical cancer is one of the most life-threatening malignancies of the reproductive system and is a significant global threat to women’s health and quality of life [1]. According to the World Health Organization (WHO), cervical cancer is the fourth most common malignant tumour in women worldwide. In 2020 alone, approximately 604,000 new cases and 342,000 deaths were reported globally [2]. The high incidence and mortality rates underscore the urgent need for effective diagnostic and therapeutic strategies to combat this disease.

Treatment options for cervical cancer include surgery, chemotherapy, radiotherapy or a combination of these [3–5]. Among these, intensity-modulated radiation therapy (IMRT) and image-guided brachytherapy have emerged as critical techniques in modern clinical practice [6–8]. These advanced radiotherapy methods enable precise delivery of therapeutic agents to cancerous tissues while minimizing damage to surrounding healthy tissues, significantly improving tumour control rates and patient survival outcomes. However, the effectiveness of these methods depends heavily on the accurate extraction and delineation of tumour regions from medical images, which remains a major challenge. In addition, inaccurate segmentation can lead to suboptimal treatment planning, resulting in insufficient irradiation of tumour tissues or unnecessary exposure of healthy organs. This not only undermines the effectiveness of the treatment but also adversely affects the patient’s prognosis.

Magnetic resonance imaging (MRI) has become the gold standard for the diagnosis and treatment of cervical cancer [9]. The MRI provides non-invasive and high-resolution imaging with excellent soft tissue contrast [10], making it an invaluable tool for assessing tumour location, shape, size, boundaries and texture. It plays a critical role in assessing the extent of tumour spread to adjacent structures such as the uterus, vagina, parametrium, bladder wall and rectal wall [11]. These advantages make MRI particularly suitable for guiding treatment planning and monitoring outcomes in cervical cancer.

However, the use of MR images for cervical tumour segmentation in clinical practice often relies on manual annotation by radiologists. While this approach provides the necessary tumour localisation and segmentation for treatment planning, it has limitations. Manual delineation is time-consuming, labour-intensive and subject to intra- and inter-observer variability. Furthermore, the segmentation process is complicated by the small volume of cervical tumours, indistinct boundaries and heterogeneous internal grey level distributions [7,12]. These challenges are exacerbated by the use of large slice thicknesses in routine hospital imaging, which reduces continuity between slices and compromises the clarity of axial, coronal and sagittal views.

In recent years, the rapid development of artificial intelligence AI and medical image analysis technology has provided innovative solutions for automated segmentation of cervical tumours based on MRI. Automated segmentation algorithms can be broadly divided into traditional methods and deep learning methods [13–15]. For traditional-based methods [16–18], these typically rely on image processing techniques such as threshold segmentation, region growing, edge detection and watershed algorithms, which achieve segmentation based on pixel intensity, boundary features or regional consistency. However, these methods are highly dependent on image quality and pre-processing, and perform poorly when faced with the complex texture characteristics and blurred boundaries of cervical tumours. In addition, their limited ability to generalise makes them insufficient to meet clinical needs. In contrast, deep learning-based methods use end-to-end learning frameworks that can automatically extract multi-level features from large datasets, significantly improving segmentation accuracy and efficiency [19,20]. For example, Unet and its variants (e.g., 3D UNet [21], ResUNet [22], TransUNet [23], SwinUNet [24], DenseUNet [25], etc.) have shown outstanding performance in medical image setmentation tasks. Despite the significant progress made by deep learning methods in automated segmentation, several challenges remain. These include a high dependence on the quantity and quality of annotated data, limited generalization across different imaging devices and parameters and insufficient exploitation of the multi-view information inherent in MRI data [26]. These limitations prevent the full exploitation of three-dimensional structural information and ultimately affect the accuracy and robustness of segmentation.

To address the above challenges, we propose a novel segmentation model based on multi-view feature transfer learning, called MVT-Net. The MVT-Net treats a 2D global axial plane encoder-decoder network as the source domain and a 3D multi-scale segmentation network as the target domain, transferring rich tumour representations across views to bolster spatial and contextual awareness. By aligning and fine-tuning features between the 2D and 3D domains, the MVT-Net can capture complementary information that neither network could learn alone. In addition, multi-scale residual blocks and multi-scale residual attention blocks are embedded in the 3D network to effectively capture feature correlations across channels and spatial locations. The MVT-Net aims to provide greater accuracy and reliability for clinical diagnosis and treatment, driving the development of cervical tumour segmentation technology.

The contributions of this paper are summarized as follows:

- We propose a novel cervical tumour segmentation model based on multi-view feature transfer learning, which integrates a 2D global axial plane encoder-decoder network and a 3D multi-scale segmentation network.
- A multi-scale residual block and a multi-scale residual attention block are proposed, which are designed to effectively capture feature correlations across channels and spatial locations, while improving the model’s ability to segment tumours with complex boundaries and small structures.
- A transfer learning strategy is used to extract tumour-related information from both 2D and 3D perspectives, integrating multi-view features to enhance the robustness and generalisation of the model.
- Our MVT-Net outperforms SOTA methods in experiments conducted on a cervical MR dataset of 160 images. In addition, ablation studies confirm the effectiveness of the proposed modules, highlighting the significant progress made in cervical tumour segmentation.

The remainder of this paper is organized as follows. Section 2 reviews the related work on cervical tumour segmentation. Section 3 introduces details of the specific structure of the proposed method. Section 4 presents and analyses the experimental results. Section 5 discusses the implications, potential limitations. Section 6 concludes this work.

## 2 Related work

### 2.1. Cervical MRI Segmentation

For traditional-based methods, Remya *et al*. [27] proposed a hierarchical adaptive local affine alignment method for simultaneous detection and segmentation of cervical cancer MR images. Arbones *et al*. [28] combined histogram-based ROI detection, level set segmentation, and morphological operations to delineate tumours and metastatic lymph nodes. Lu *et al*. [29] introduced a normalized Bayesian framework for non-rigid registration and tumour probability mapping. Torheim *et al*. [30] employed Fisher’s linear discriminant analysis with voxel-level features for cervical tumour segmentation. Khoulqi *et al*. [31] developed a multi-stage method incorporating K-means de-noising, region growing, and FIGO-based staging. Berendsen *et al*. [32] added a statistical regularization term to deformable registration to reduce local minima. Su *et al*. [33] proposed a globally adaptive region growing algorithm to enhance boundary extraction in MRI-based cervical tumour segmentation.

However, these traditional methods are often limited by complex processing steps, poor generalization, high computational cost, and heavy dependence on manual feature engineering, restricting their clinical applicability. To overcome these limitations and meet the growing demands for accuracy and efficiency, deep learning-based approaches have emerged as a powerful alternative, offering automatic feature extraction and superior segmentation performance.

Deep learning-based segmentation methods have significantly advanced radiotherapy for cervical cancer. Ju *et al*. [34] employed Dense V-Net for CT-based CTV delineation with small samples. Kano *et al*. [35] trained 2D and 3D U-Nets independently, generating final segmentation via binarization of multiple predictions. Lin *et al*. [9] combined U-Net and radiomics features to segment cervical tumour MR images. Bnouni *et al*. [36] introduced a GAN-based method integrating cross-sectional and sagittal MR images to improve spatial consistency. Yoganathan *et al*. [37] proposed 2D and 2.5D residual networks for automatic contouring in HDR brachytherapy. Gou *et al*. [38] developed a multi-view feature attention network to address challenges like intensity inhomogeneity and 3D contextual limitations. Huang *et al*. [39] optimized CNNs with attention mechanisms for automated OAR segmentation from multi-sequence MRIs. Wang *et al*. [40] presented a 3D CNN for multimodal MRI-based lesion identification, improving segmentation accuracy and convergence speed. Collectively, these studies demonstrate the potential of deep learning to enhance cervical tumour segmentation and promote clinical integration.

### 2.2. Transfer learning on medical images

The goal of transfer learning is to leverage knowledge from one or more source domains to improve model performance in the target domain. Given the limited availability of medical image data and the challenges in accurately delineating tumour markers, transfer learning has become widely used in medical image segmentation tasks [41]. Researchers have extensively explored domain adaptation techniques within transfer learning to bridge the gap between source and target domains, enabling better performance across diverse datasets. For example, Agarwal *et al*. [42] applied transfer learning to a convolutional neural network (CNN) model for lesion segmentation in small gastrointestinal datasets, achieving promising results. Similarly, Chen *et al*. [43] used pre-trained InceptionV3 and VGG-16 models from the ImageNet dataset and fine-tuned them for prostate cancer MR image segmentation, demonstrating that the VGG-16 model, through transfer learning, outperformed traditional methods in segmenting prostate cancer MR images.

While these studies highlight the versatility of transfer learning across various domains, its potential in the context of cervical tumour MR image segmentation is particularly noteworthy. By transferring knowledge from large, general medical image datasets or related organ segmentation tasks, transfer learning can significantly enhance cervical tumour segmentation performance. In fact, several studies have fine-tuned pre-trained models from natural image datasets to medical image datasets, improving feature extraction efficiency and segmentation accuracy. For instance, Lin *et al*. [44] employed DeepLab V3, initially trained on a general cervical cancer dataset, and fine-tuned the network for cervical tumour segmentation. This approach highlights the effectiveness of transfer learning in adapting pre-trained models for specialized medical tasks.

Building on this, our study further explores the application of transfer learning to cervical tumour MR image segmentation. Specifically, we propose a novel multi-view feature transfer learning method aimed at addressing the current challenges and improving segmentation performance.

## 3 Method

**Fig 1:**
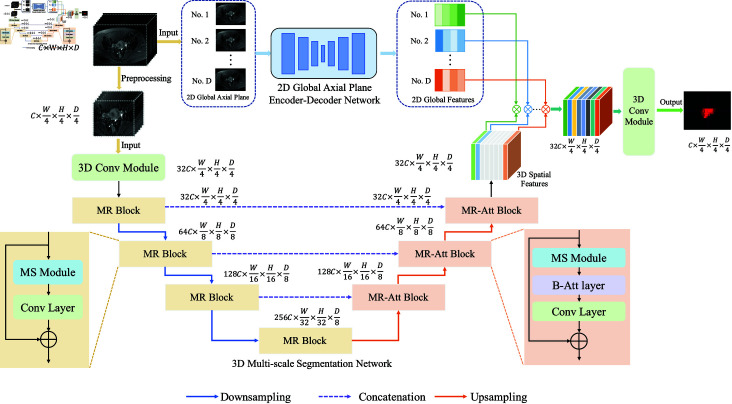
The overall architecture of the proposed MVT-Net.

To address the insufficient utilization of multi-view information inherent in MRI data, we propose a cervical tumour segmentation model based on multi-view feature transfer learning (MVT-Net), as shown in Figure 1. The MVT-Net consists of two main components: a 2D global axial plane encoder-decoder network as the source domain and a 3D multi-scale segmentation network as the target domain. This approach uses a transfer learning strategy to fully extract and integrate diverse knowledge about cervical tumours from multiple perspectives.

Within the 3D multi-scale segmentation network, we incorporate a multi-scale residual block (MR) and a multi-scale residual attention block (MR-Att) to effectively capture feature correlations across channels and spatial dimensions. The MR block enables the extraction of features at various scales, allowing the network to recognize both fine details and large tumour structures. Meanwhile, the MR-Att block adaptively assigns higher weights to the most informative features, helping the model concentrate on critical tumour regions while suppressing irrelevant or redundant information. Together, these modules enhance the network’s ability to manage the complex morphology and heterogeneity of cervical tumours.

In addition, the multi-view feature transfer learning Module serves as a critical link between the 2D source view and the 3D target view. By transferring the rich feature representations learned from 2D axial images to the 3D segmentation task, this module allows the network to fully utilize complementary information from both views. It effectively bridges the gap between the global contextual information captured in 2D images and the localized spatial details required for accurate 3D segmentation, ensuring a seamless fusion of multi-view features.

By integrating these multi-view features and leveraging the strengths of each module, MVT-Net achieves a more comprehensive understanding of cervical tumour characteristics. This integrated approach not only improves segmentation accuracy by accounting for complex tumour boundaries and heterogeneity, but also increases the robustness of the model under varying imaging conditions.

### 3.1. Multi-scale residual and multi-scale residual attention block

The 3D multi-scale segmentation network incorporates a multi-scale residual block in the encoder part of the network. This block integrates residual learning with a multi-scale feature representation module, allowing effective extraction of hierarchical features. In the decoder section, a multi-scale residual attention block is used, which combines a multi-scale feature representation module with a bottleneck attention mechanism. This design enhances the network’s ability to focus on critical features while preserving essential spatial and contextual information.

#### 3.1.1. Multi-scale residual block.

**Fig 2:**
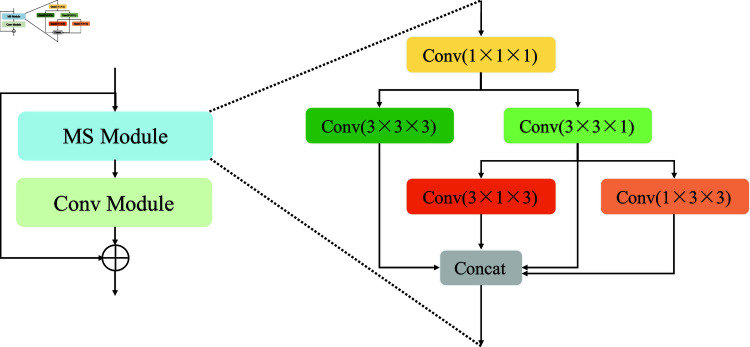
The detailed structures of multi-scale residual block.

Taking into account the features of T2-weighted MR image data, the residual module theory is integrated with a multi-scale module [38] in the encoder part of the segmentation network, resulting in the development of a multi-scale residual block (MR Block), as shown in Fig 2. To improve the network’s ability to capture complex feature patterns, the feature map from the previous stage is first processed through a 1×1×1 convolution layer. The output is then split into two main branches. The upper branch applies a 3×3×3 convolution to capture comprehensive 3D spatial features. The lower branch focuses on plane-specific feature extraction: it uses 3×3×1 convolutions along the axial plane, where higher resolution facilitates the delineation of tumours and tissues, and employs 3×1×3 and 1×3×3 convolutions to extract complementary features from the sagittal and coronal planes. Finally, features from the 3D and three 2D planes are fused along the channel dimension, providing a rich multi-scale representation to support cervical tumour image decoding.

#### 3.1.2. Multi-scale residual attention block.

**Fig 3:**
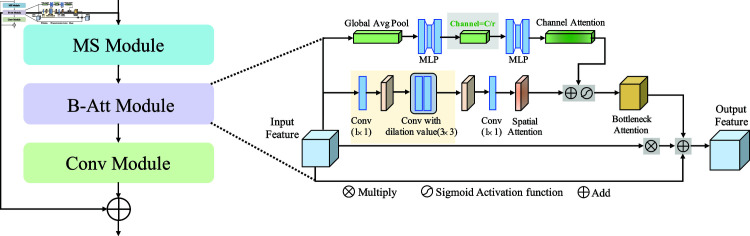
The detailed structures of multi-scale residual attention block.

Inspired by the attention mechanism [45], this paper introduces the multi-scale residual attention block (MR-Att Block) in the decoding part of the network. This block consists of the multi-scale feature representation module and the bottleneck attention module, as shown in Fig 3. The MR-Att Block enhances the network’s ability to interpret feature relationships at different scales, allowing it to adapt more effectively to complex task requirements. Within this block, the bottleneck attention module (B-Att Module) utilizes two branches: channel attention and spatial attention. The channel attention branch processes feature information along the channel dimension, while the spatial attention branch focuses on feature relationships across spatial dimensions. Together, these branches enable the network to selectively emphasize or suppress features, performing fine-tuning on intermediate feature representations to strengthen the network’s overall feature extraction capabilities. The resulting channel attention map and spatial attention map are then combined to produce a final 3D attention map. This process allows the network to simultaneously capture feature correlations across channels and spatial information from different locations, ultimately improving its performance in complex segmentation tasks.

### 3.2. Multi-view feature transfer learning

**Fig 4:**
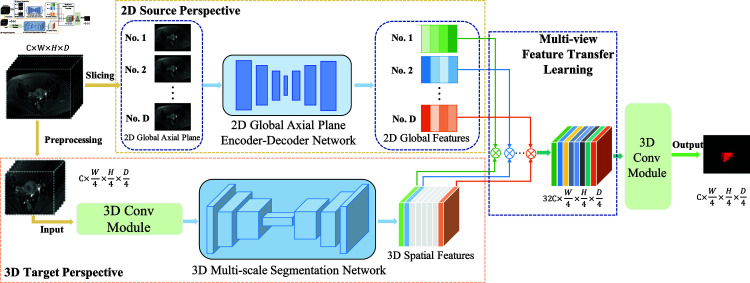
The Architecture of multi-view feature transfer learning.

The multi-view feature transfer module integrates a 2D source perspective with a 3D target perspective, as shown in Fig 4. The primary objective of this module is to transfer axial cervical tumour images from the 2D source perspective to the spatial features of the cervical tumour in the 3D target perspective. The features of 2D source perspective and 3D target perspective are extracted using 2D and 3D convolution operations, respectively. The core function of the multi-view feature transfer module is to effectively merge information from different perspectives, facilitating a comprehensive understanding of the morphology and spatial positioning of cervical tumours. This integration significantly improves the accuracy and robustness of cervical tumour segmentation.

### 3.3. Training objective

To address the imbalance between target and background in cervical tumour MR images, as well as the problem of scattered false-positive tumours in the background area, the loss function is designed by combining the dice coefficient loss and the cross entropy loss, defined as follows:

$$ℒ=\alpha {ℒ}_{dice}(T,S)+(1-\alpha ){ℒ}_{ce}(T,S),$$ (1)

where ${ℒ}_{dice}(T,S)$ is the dice coefficient loss, ${ℒ}_{ce}(T,S)$ represents the cross entropy loss and ℒ is the joint loss. The parameter α serves as a balancing factor, acting as a hyperparameter to adjust the contributions of the dice coefficient loss ${ℒ}_{dice}(T,S)$ and the cross-entropy loss ${ℒ}_{ce}(T,S)$. The formulation of the dice coefficient loss is as follows:

$$L_{dice}\left(T,S\right)=1-2\frac{\sum {\mathrm{\backslash nolimits}}_{d}^{D}\sum {\mathrm{\backslash nolimits}}_{w}^{W}\sum {\mathrm{\backslash nolimits}}_{h}^{H}T(d,w,h)S(d,w,h)}{\sum {\mathrm{\backslash nolimits}}_{d}^{D}\sum {\mathrm{\backslash nolimits}}_{w}^{W}\sum {\mathrm{\backslash nolimits}}_{h}^{H}T(d,w,h)+S(d,w,h)},$$ (2)

where *T*(*d*, *w*, *h*) denotes the ground truth and *S*(*d*, *w*, *h*) represents the segmentation result. *D*, *W* and *H* denote the dimensions of the image. The formulation for the cross entropy loss is as follows:

$$L_{ce}\left(T,S\right)=-\frac{1}{N}\sum_{i}\sum_{c=1}^{C}sign(T_{i},S_{i})\log(p_{ic}),$$ (3)

where *T*_*i*_ indicates the ground truth for the *i*-th sample and *S*_*i*_ is the segmentation result for the *i*-th sample. *C* is the number of categories, set to 2, representing the target and the background. *N* denotes the total number of samples and *p*_*ic*_ denotes the probability of segmenting the *i*-th sample as the target area. The sign(·) function is used to indicate the segmentation result. If the segmentation result for the *i*-th sample matches the target area, sign(·) is set to 1; otherwise it is set to 0.

## 4 Experiments

### 4.1. Ethical statement

This study was approved by Key Lab of Intelligent Perception and Image Understanding of Ministry of Education, by AI-based Big Medical Imaging Data Frontier Research Center, Academy of Advanced Interdisciplinary Research, Xidian University, and by the institutional review board (IRB) of First Affiliated Hospital of Air Force Medical University, China. The doctors obtained signed informed consent forms from all selected patients prior to the routine clinical course of MR examinations. For the research on cervical tumour segmentation, the Medical Ethics Committee of the First Affiliated Hospital of Air Force Medical University approved this research to access and use the dataset from December 22, 2023.

### 4.2. Dataset and preprocessing

**Fig 5:**
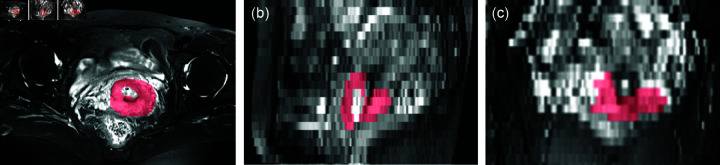
Three-view images showing the tumor location in T2-weighted MRI of a cervical cancer patient. (a) Axial image and label. (b) Sagittal image and label. (c) Coronal image and label.

The data were obtained from T2-weighted MRI of cervical cancer patients collected by the radiology department of a hospital. A total of 160 cervical tumour MRI sets were acquired from 160 patients. These images were acquired using a Siemens 3T MR scanner (TrioTim, Siemens, Erlangen, Germany) with scanning parameters including a repetition time of 5750 milliseconds and an echo time of 95 milliseconds. The image resolution ranges from 0.5 to 1.25 mm, with a slice thickness of 6 mm. Fig 5 shows the three-view T2-weighted MRI of a patient, including axial, coronal and sagittal views. It can be seen that the axial images have relatively high resolution, whereas the coronal and sagittal views appear more blurred. This variation arises from the relatively thick scanning layer used in magnetic resonance imaging, resulting in differences in the level of detail and information presented across the axial, coronal and sagittal planes.

In order to minimize the effect of the bias field on imaging, this study uses the N4ITK bias field correction to pre-process the data. Since the resolution of cervical tumour images varies, which can significantly affect the segmentation performance of deep neural network models, linear interpolation and nearest neighbour interpolation are used to resample the cervical tumour images and their corresponding labels to ensure consistent resolution across the dataset. Although resampling ensures uniform resolution for all 3D cervical tumour images, image sizes may still vary. Most 3D images contain large areas of invalid black regions, which exacerbate the imbalance between positive and negative samples in individual cases. To address this, connected components are detected to determine the maximum volume coordinates of the abdominal area and the largest circumscribed cube is extracted. Based on the centre point of this cube, a region of size 512×512×16 is cropped, and the corresponding labels are cropped to the same region. Due to GPU memory limitations, the MR 3D cervical tumour images are further processed using a sliding-window approach to generate smaller cubic blocks of size 128×128×16 for training and testing in the 3D target view domain. To evaluate the effectiveness of the proposed method, a five-fold cross-validation strategy is employed. The dataset of 160 patients is randomly divided into five groups, with one group serving as the test set and the remaining four groups as the training set.

### 4.3. Implementation details

In this work, the experiments were conducted on a desktop computer equipped with an Intel(R) Core(TM) i7-6900K CPU @ 3.20 GHz × 8, 64 GB of memory and an Nvidia TITAN X 12 GB GPU. The deep neural network model was implemented using Python 3.7 and the TensorFlow 1.14.0 framework, with CUDA 12.2 and CUDNN 7.6.5 libraries for GPU acceleration. During the training process, the initial learning rate was set to 0.0001. The learning rate was halved if the validation loss did not decrease for 2 consecutive epochs. Training was terminated if the validation loss did not improve for 4 consecutive epochs, ensuring efficient and optimized training for each stage.

### 4.4. Comparison methods

To evaluate the effectiveness of our proposed MVT-Net, we performed comparative experiments using three established segmentation models: U-Net, ResNet and GC-Net.

- U-Net [46]: It is recognised as the benchmark model for medical image segmentation and uses an encoder-decoder architecture with skip links, allowing accurate segmentation even with limited training data. Its strong ability to extract multi-scale features establishes it as a reliable baseline for medical imaging tasks.
- ResNet [47]: It is designed to overcome the challenges associated with deeper architectures, such as gradient vanishing, gradient explosion, overfitting and negative optimization. By incorporating residual connections, Res-Net facilitates efficient gradient flow, enabling the training of very deep networks while maintaining high segmentation accuracy.
- GC-Net [48]: It employs a global context network utilizing the squeeze-and-excitation (SE) module to capture global contextual information. This approach enhances the network’s representational power by focusing on features critical to segmentation tasks while reducing computational complexity without compromising accuracy.

### 4.5. Evaluation metrics

To evaluate the proposed MVT-Net and compared methods, five evaluation metrics are used to assess and compare the segmentation performance:

(1) Dice similarity coefficient (Dice):
  $$Dice=\frac{2\times \|V_{A}\cap V_{B}\|}{\|V_{A}\|+\|V_{B}\|},$$ (4)
(2) Sensitivity (SEN):
  $$SEN=\frac{\|V_{A}\cap V_{B}\|}{\|V_{A}\|},$$ (5)
(3) Positive prediction value (PPV):
  $$PPV=\frac{\|V_{A}\cap V_{B}\|}{\|V_{B}\|},$$ (6)
(4) Average surface distance (ASD):
  $$ASD=\frac{1}{2}\times \{\frac{\sum {\mathrm{\backslash nolimits}}_{Z\in S_{B}}d\left(z,S_{A}\right)}{|S_{B}|}+\frac{\sum {\mathrm{\backslash nolimits}}_{Z\in S_{A}}d\left(z,S_{B}\right)}{|S_{A}|}\},$$ (7)
(5) 95% maximum surface distance (95SD):
  $$95SD=\frac{1}{2}\times \left[K_{95}\underset{Z\in S_{B}}{min}d\left(z,S_{A}\right)+K_{95}\underset{Z\in S_{A}}{min}d\left(z,S_{B}\right)\right],$$ (8)
where $V_{A}$ denotes the set of ground truth, $V_{B}$ represents the set of segmentation results, *S*_*A*_ refers to the voxels in the ground truth, and *S*_*B*_ refers to the voxels in the segmented organ surface. The terms $d\left(z,S_{A}\right)$ and $d\left(z,S_{B}\right)$ represent the minimum Euclidean distance of voxel $z\in S_{A}$ and $z\in S_{B}$ to the nearest voxels in *S*_*A*_ and *S*_*B*_, respectively. *K*_95_ denotes the 95% quantile, taking the maximum value from the first 95% of the distances.

### 4.6. Segmentation results

Table 1 shows a comparison of the segmentation performance between the proposed method and the baseline methods, U-Net, Res-Net and GC-Net on the 2D cervical tumour MRI dataset. It is evident that the multi-view feature transfer-based cervical tumour segmentation (MVT-Net) considerably outperforms U-Net, Res-Net and GC-Net in terms of segmentation accuracy and tumour recognition. The results demonstrate that the MVT-Net excels on several critical aspects of cervical tumour segmentation, including precise localisation, accurate shape representation, clear edge delineation and a comprehensive understanding of the spatial context of the tumour. These significant improvements highlight the superior performance of the MVT-Net and provide robust evidence for the efficacy of the multi-view feature transfer strategy and the multi-scale attention mechanism. Based on these advanced techniques, the MVT-Net is able to effectively capture the complex characteristics of cervical tumours, ultimately leading to more accurate and reliable segmentation results.

**Table 1:** Comparison of experimental results for cervical tumour segmentation algorithms.

Methods	Dice (%)	SEN (%)	PPV (%)	ASD (mm)	95SD (mm)
U-Net	63.3±16.7	56.5±22.5	80.9±10.0	3.47±1.48	14.24±8.50
Res-Net	66.1±18.1	61.0±22.7	80.3±10.3	3.23±1.28	14.06±7.35
GC-Net	71.8±11.5	70.7±18.1	77.4±11.8	2.97±1.05	12.97±6.64
**MVT-Net**	**75.9 ± 7.43**	**76.5 ± 11.5**	**81.2 ± 10.4**	**2.69 ± 0.58**	**10.84 ± 3.69**

**Fig 6:**
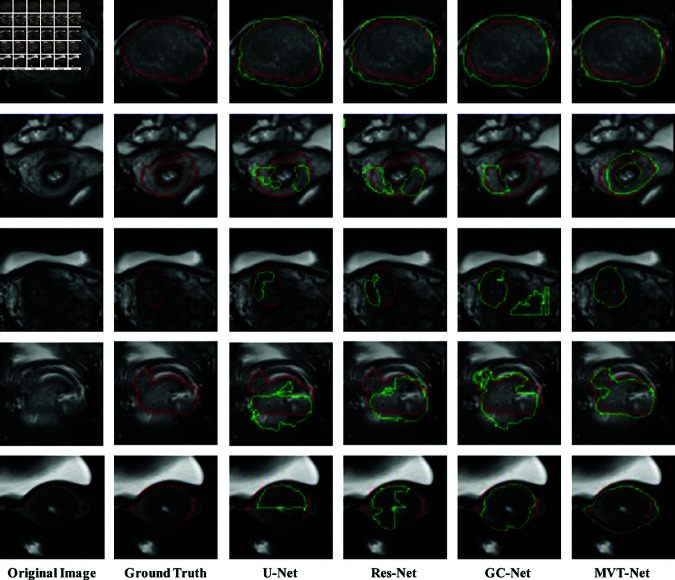
Comparison of experimental results of 2D cervical tumour segmentation networks.

To visually demonstrate the performance of our proposed MVT-Net and the comparison methods U-Net, Res-Net and GC-Net in the cervical tumour segmentation task, we visualized the segmentation results on the axial 2D plane as shown in Fig 6. The red box represents the ground truth of the cervical tumour, while the green box shows the experimental segmentation results.

The MRI segmentation results for cervical tumours shown in Fig 6 show that for larger tumours, all four segmentation models—U-Net, Res-Net, GC-Net and MVT-Net—produce reasonably accurate segmentation results. However, the first three models (U-Net, Res-Net and GC-Net) tend to oversegment the tumour boundaries to varying degrees by including additional tissue areas that are not part of the tumour. This over-segmentation is most noticeable along the tumour’s edges, where the models incorrectly classify surrounding tissues as part of the tumour. For small volume tumours, U-Net and Res-Net show under-segmentation and missed segmentation in several cases, particularly in the third, fourth and fifth rows. These models fail to capture significant portions of the tumour, resulting in the omission of critical tumour information and incomplete segmentation. Such under-segmentation can be particularly problematic in clinical settings, where accurate tumour delineation is critical for diagnosis and treatment planning. While GC-Net generally performs well, it occasionally mis-segments certain regions, leading to inaccuracies in the tumour boundaries. These mis-segments can affect overall segmentation quality, particularly in regions where tumour boundaries are less well defined. In contrast, our proposed MVT-Net demonstrates superior performance, especially in segmenting small tumours. It achieves more accurate tumour localization and provides clearer, more precise boundary delineation. The improved segmentation accuracy can be attributed to the multi-view feature transfer strategy and the multi-scale attention mechanism, which enable MVT-Net to capture both global context and fine-grained details of the tumour. This makes MVT-Net particularly effective in challenging cases involving small or irregularly shaped tumours.

**Fig 7:**
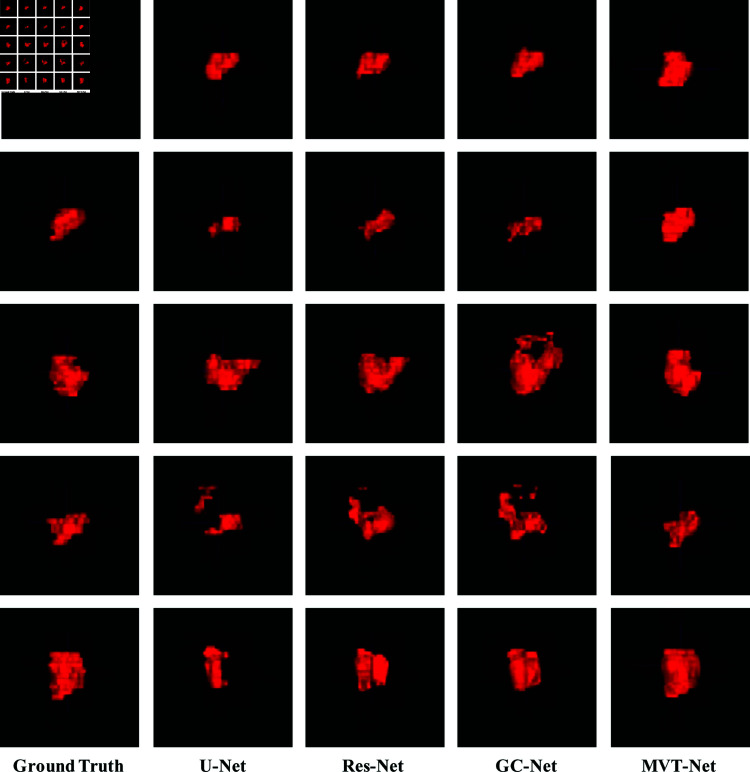
Comparison of experimental results of 3D cervical tumour segmentation networks.

Fig 7 shows the results of the comparative experiment on 3D cervical tumour segmentation. It can be observed that the 3D segmentation results produced by U-Net, Res-Net and GC-Net show some scattered points, reflecting the limitations of these methods in effectively capturing tumour and tissue information from the axial sections of cervical tumour MR images. In contrast, our proposed MVT-Netuses a multi-scale attention mechanism and a multi-dimensional view feature transfer strategy. This approach enables the comprehensive extraction of both 2D and 3D views together with contextual information, providing enriched feature representations for the 3D cervical tumour segmentation network. As a result, the MVT-Net model demonstrates significant improvements in 3D cervical tumour segmentation accuracy and quality.

### 4.7. Ablation analysis

Table 2 shows the results of our proposed MVT-Net and comparison methods on cervical tumour MRI data across five evaluation metrics. As shown in Table 2, our proposed MVT-Net achieves significant improvements in segmentation performance metrics compared to MF-Net, RCA-Net and MRCA-Net. Although the standard deviation of our proposed MVT-Net is slightly higher (0.16 mm) than that of MRCA-Net, its overall performance is more stable, with relatively less variation in performance. This indicates that by transferring axial cervical tumour image features from the 2D source view to the spatial features of the 3D target view, our proposed MVT-Net effectively improves segmentation results for 3D cervical tumours, demonstrating better segmentation performance across all evaluation metrics.

**Table 2:** Results of the ablation experiments for cervical tumor segmentation.

MF	RCA	MV-Transfer	Methods	Dice(%)	SEN(%)	PPV(%)	ASD(mm)	95SD(mm)
✓	✗	✗	MF-Net	67.6±15.6	63.5±9.8	79.3±10.3	3.35±1.21	13.90±7.10
✗	✓	✗	RCA-Net	70.7±12.6	67.6±18.4	78.8±9.6	2.86±0.77	12.39±5.26
✓	✓	✗	MRCA-Net	74.4±10.4	73.1±16.3	78.6±8.3	2.53 ± 0.77	11.18±5.87
✓	✓	✓	MV-TNet	**75.9 ± 7.43**	**76.5 ± 11.5**	**81.2 ± 10.4**	2.69 ± 0.58	**10.84 ± 3.69**

**Fig 8:**
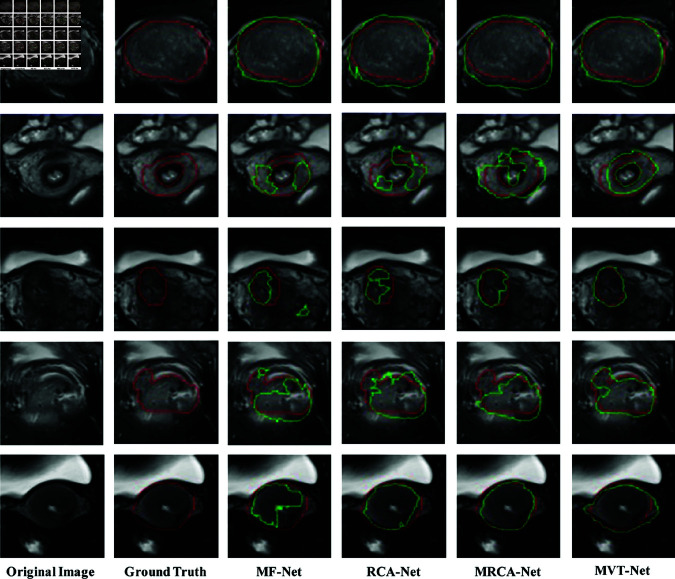
Results of ablation experiments of 2D cervical tumour segmentation networks.

To validate the effectiveness of the proposed MVT-Net and its components, MF-Net, RCA-Net and MRCA-Net, a visual analysis of their 2D segmentation results was performed, as shown in Fig 8. In the figure, the red box represents the ground truth labels of cervical tumour MR images, while the green box represents the experimental segmentation results. From Fig 8, it can be observed that for larger tumours, all four methods—MF-Net, RCA-Net, MRCA-Net and MVT-Net can effectively detect and segment the tumours. However, MF-Net, RCA-Net and MRCA-Net exhibit varying degrees of over-segmentation along the tumour boundaries. In contrast, MVT-Net demonstrates excellent segmentation performance in regions with strong boundary contrast, accurately distinguishing tumours from normal tissue. However, its performance in regions with blurred boundaries still requires further improvement. For cases with smaller tumour areas, MF-Net, RCA-Net and MRCA-Net show different levels of mis-segmentation and under-segmentation, as observed in the third, fourth and fifth rows of Fig 8. Specifically, MF-Net produces false positive regions, RCA-Net loses a significant amount of information about the tumour region, while MRCA-Net, which incorporates multi-view features and attention mechanisms, outperforms the first two methods. In general, MVT-Net exhibits more balanced segmentation performance, achieving high accuracy in both large and small tumour segmentation tasks. However, its robustness in handling tumours with blurred boundaries still needs further enhancement. These observations highlight the superiority of the multi-view transfer strategy used in the MVT-Net method. In particular, this strategy demonstrates its effectiveness in dealing with small area tumours, providing more accurate and clearly defined segmentation results.

**Fig 9:**
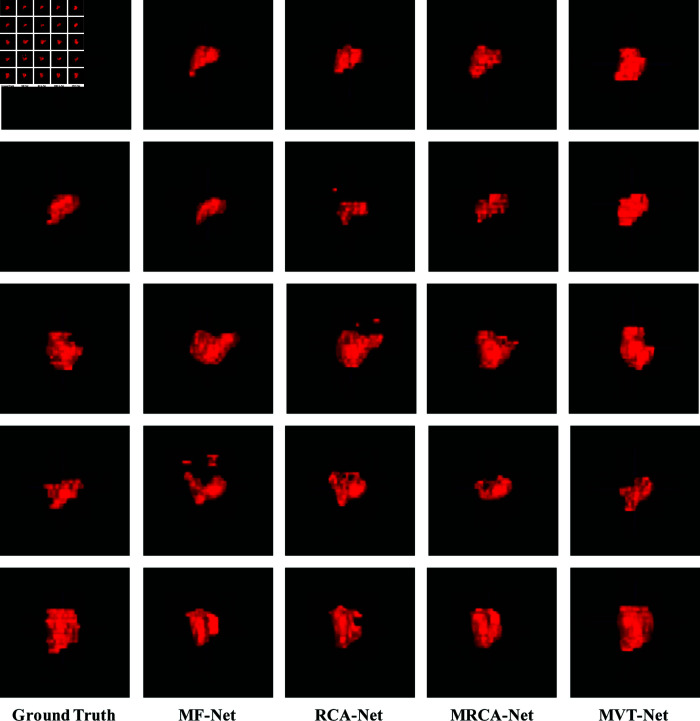
Results of ablation experiments of 3D cervical tumour segmentation networks.

Fig 9 shows the results of ablation experiments with five sets of 3D cervical tumour segmentation networks. In the 3D cervical tumour segmentation results produced by the MF-Net and RCA-Net methods, some discrete points are noticeable. In contrast, the MRCA-Net method shows superior performance in suppressing these discrete segmentation artefacts and achieves more accurate localisation of the lesion area. In particular, the proposed MVT-Net effectively extracts valuable feature information from the axial section images of the 2D source view, providing robust feature support for the 3D cervical tumour segmentation network. This capability significantly improves the performance of MVT-Net in 3D cervical tumour segmentation tasks. However, due to the blurred boundaries between tumour and non-tumour regions during the information transfer process of 2D cervical tumour images, the network may occasionally misclassify some non-tumour axial section images as tumour regions, resulting in a small number of false positives in the 3D segmentation results. These observations further highlight the advantages of the multi-view feature transfer strategy employed in the MVT-Net approach, in particular its critical role in providing rich and robust feature information support. However, they also suggest that further refinement and optimization is required to effectively minimise the occurrence of false positives in 3D cervical tumour segmentation tasks.

## 5 Discussion

For the multi-scale residual and multi-scale bottleneck attention mechanisms, they effectively extract multi-scale feature information from cervical images while reducing the computational complexity and improving the non-linear fitting ability of the network. In the decoder, the bottleneck attention module generates channel and spatial attention maps, which are fused into a 3D attention map. This enables the network to capture both channel-wise and spatial dependencies, improving adaptability and segmentation precision. Experimental results show that the multi-scale residual and multi-scale bottleneck attention mechanisms effectively mitigate problems such as gradient vanishing and gradient explosion in deep learning networks. Furthermore, they significantly improve the efficiency and overall performance of the network, providing reliable support for cervical tumour segmentation tasks.

In terms of the multi-view feature transfer learning strategy, the cervical tumour segmentation method based on multi-view feature transfer consists of two branches: a 2D source view network and a 3D target view network. This strategy transfers the axial cervical tumour image features from the 2D source view to the spatial features of the cervical tumour in the 3D target view, which ultimately produces the 3D cervical tumour segmentation results. Experimental results show that this strategy effectively exploits information from different views, significantly improving the accuracy and robustness of cervical tumour segmentation and improving the overall performance of the segmentation task.

Regarding the design of the loss function, we design a total loss function that combines coefficient loss and cross-entropy loss to efficiently train the cervical tumour segmentation network based on multi-view feature transfer. In addition, a balancing factor is introduced as a hyperparameter to regulate the contributions of these two loss components during the training process. Experimental results show that this loss function effectively mitigates the problem of scattered false positives in the background regions of cervical tumour MRI images during 3D tumour segmentation. As a result, it significantly improves segmentation accuracy and overall performance in cervical tumour segmentation tasks.

In summary, the multi-scale attention segmentation method based on multi-view feature transfer has significantly improved the performance of cervical tumour detection and segmentation. However, from the 2D image segmentation results, there are still cases where the tumour edges are partially biased towards the background region, especially in the segmentation results of ring-shaped cervical tumour images. This problem is mainly caused by the imbalance between background pixels and tumour pixels in cervical tumour MRI images, which causes the training model to fall into a local optimum. To address challenges such as the variability of cervical tumour shapes and the inaccuracy of edge segmentation, future research could focus on optimising and improving the loss function for cervical tumour segmentation. This would further improve the segmentation accuracy and refine the overall segmentation results.

## 6 Conclusion

In this paper, we propose a novel cervical tumour segmentation network model (MVT-Net) based on a multi-view feature transfer strategy that integrates a 2D global axial plane encoder-decoder network and a 3D multi-scale segmentation network to extract 2D and 3D view information from MRI images. The multi-scale residual block and the multi-scale residual attention block are introduced into the segmentation network to effectively capture feature correlations across channels and spatial locations, significantly improving the model’s performance in handling complex boundaries and small tumours. In addition, a transfer learning strategy is designed to integrate multi-view features, further enhancing the model’s robustness and generalisability. Experimental results show that the proposed method achieves excellent performance and strong ability to generalisation in cervical tumour segmentation tasks. However, MVT-Net has certain limitations, i.e., when transferring 2D cervical tumour information, some non-tumour regions may be incorrectly segmented as tumour regions. Future research can focus on optimizing and improving the loss function for cervical tumour segmentation to further improve segmentation accuracy and refine the overall segmentation performance.
